# Supplementary material for: MaAsLin 3: Refining and extending generalized multivariable linear models for meta-omic association discovery
Source: bioRxiv. 2024 Dec 14:2024.12.13.628459. Preprint. [Version 1] doi: 10.1101/2024.12.13.628459 (PMC11661281; doi:10.1101/2024.12.13.628459)
Supplement: 1 [file NIHPP2024.12.13.628459V1-supplement-1.pdf]

bioRxiv preprint doi: https://doi.org/10.1101/2024.12.13.628459; this version posted December 14, 2024. The copyright holder for this preprint (which was not certified by peer review) is the author/funder, who has granted bioRxiv a license to display the preprint in perpetuity. It is made available under aCC-BY 4.0 International license.

Supplementary Information

Relative and absolute coefficients differ by a constant shift when extraction efficiency is equal and all features are present

First, consider the case in which all features have equal extraction efficiency and there is no sparsity. Following the notation of ANCOM-BC, let $i \in \{1, ..., m\}$ be the feature index, $j \in \{1, ..., g\}$ be the covariate index, and $k \in \{1, ..., n\}$ be the sample index. Let $\mathbf{X}$ be the $n \times (g+1)$ design matrix of the metadata (including the intercept). With A_{ik} as the absolute abundance of feature i in sample k, let Z_{ik} be the $\log_2$ absolute abundance, so $Z_{ik} = \log_2(A_{ik})$. Also, suppose the absolute abundance is related to the metadata by: $E(Z_{ik}) = \sum_j X_{kj}\beta_{ij} + \epsilon_{ik}$ where β_{ij} is the slope relating covariate j to feature i's $\log_2$ absolute abundance and ϵ_{ik} is some error with mean 0. These slopes can be grouped together for a feature as a $(g+1) \times 1$ column vector: $\boldsymbol{\beta}_i = (\beta_{i0}, \beta_{i2}, ..., \beta_{ig})^T$. Let the $n \times 1$ vector $\mathbf{Z}_i = (Z_{i1}, Z_{i2}, ..., Z_{in})^T$ denote the vector of $\log_2$ absolute abundances for feature i. With $T_k = \sum_i A_{ik}$ as the total absolute abundance in sample k, let $D_k = \log_2(T_k)$. With $P_{ik} = A_{ik}/T_k$ as the relative abundance of feature i in sample k, let $Y_{ik} = \log_2(P_{ik})$, so $2^{Y_{ik}} = 2^{Z_{ik}}/2^{D_k} \iff Y_{ik} = Z_{ik} - D_k$. As with the absolute abundances, let $\mathbf{Y}_i$ be the $n \times 1$ vector of $\log_2$ relative abundances for feature i, and let $\mathbf{D}$ be the $n \times 1$ vector of $\log_2$ total abundances. Thus, $\mathbf{Y}_i = \mathbf{Z}_i - \mathbf{D}$ for all i.

When observing a vector $\mathbf{Z}_i$ and wanting to estimate $\boldsymbol{\beta}_i$, the ordinary least squares method is typically used: $\hat{\boldsymbol{\beta}}_i = (\mathbf{X}^T\mathbf{X})^{-1}\mathbf{X}^T\mathbf{Z}_i$. By the abundance decomposition above, this also gives:

$$\hat{\boldsymbol{\beta}}_i = (\mathbf{X}^T\mathbf{X})^{-1}\mathbf{X}^T\mathbf{Z}_i = (\mathbf{X}^T\mathbf{X})^{-1}\mathbf{X}^T\mathbf{Y}_i + (\mathbf{X}^T\mathbf{X})^{-1}\mathbf{X}^T\mathbf{D} = \hat{\boldsymbol{\beta}}_i^{\text{rel}} + \hat{\boldsymbol{\beta}}^{\text{tot}}$$

where $\hat{\boldsymbol{\beta}}_i^{\text{rel}}$ is the result of regressing the $\log_2$ relative abundances on the design matrix, and $\hat{\boldsymbol{\beta}}^{\text{tot}}$ is the result of regressing the $\log_2$ total abundances on the design matrix. Note that since $\hat{\boldsymbol{\beta}}^{\text{tot}}$ is the same for all features, if the absolute abundance coefficient for one feature i is d larger than the absolute abundance coefficient for another feature i' (i.e., $\hat{\beta}_{ij} - \hat{\beta}_{i'j} = d$), the relative abundance coefficient for feature i will be d larger than the relative abundance coefficient for feature i' (i.e., $\hat{\beta}_{ij}^{\text{rel}} - \hat{\beta}_{i'j}^{\text{rel}} = d$). Thus, if all that is available is the relative abundance data, absolute slopes $\hat{\beta}_{ij}$ themselves cannot be determined. However, the relative coefficients $\hat{\beta}_{ij}^{\text{rel}}$ *can* be determined, and the ordering of and spacing between these coefficients will be identical to the ordering of and spacing between the absolute coefficient for each metadatum.

Since the OLS solution is unbiased for $\boldsymbol{\beta}_i$, the expectations will be equal too:

$$\boldsymbol{\beta}_i = E(\hat{\boldsymbol{\beta}}_i) = E(\hat{\boldsymbol{\beta}}_i^{\text{rel}}) + E(\hat{\boldsymbol{\beta}}^{\text{tot}}) = \boldsymbol{\beta}_i^{\text{rel}} + \boldsymbol{\beta}^{\text{tot}}.$$

Assuming at least half of the features do not change with respect to a particular metadatum (i.e., $\beta_{ij} = 0$ for at least half the features i), the median absolute abundance coefficient will be 0 (i.e., $\text{med}(\beta_{1j}, \beta_{2j}, ..., \beta_{mj}) = 0$), as has been previously noted in LOCOM.[31] Expanding each of these coefficients using the expectation decomposition above gives

$$\text{med}(\beta_{1j}^{\text{rel}} + \beta_j^{\text{tot}}, \beta_{2j}^{\text{rel}} + \beta_j^{\text{tot}}, ..., \beta_{mj}^{\text{rel}} + \beta_j^{\text{tot}}) = 0.$$

Since the term β_j^{tot} is the same in all of these, this implies $\text{med}(\beta_{1j}^{\text{rel}}, \beta_{2j}^{\text{rel}}, ..., \beta_{mj}^{\text{rel}}) = -\beta_j^{\text{tot}}$. Thus, a test of $\beta_{ij} = 0$ is algebraically equivalent to the tests:

$$\beta_{ij}^{\text{rel}} + \beta_j^{\text{tot}} = 0 \iff \beta_{ij}^{\text{rel}} = \text{med}(\beta_{1j}^{\text{rel}}, \beta_{2j}^{\text{rel}}, ..., \beta_{mj}^{\text{rel}}).$$

bioRxiv preprint doi: https://doi.org/10.1101/2024.12.13.628459; this version posted December 14, 2024. The copyright holder for this preprint (which was not certified by peer review) is the author/funder, who has granted bioRxiv a license to display the preprint in perpetuity. It is made available under aCC-BY 4.0 International license.

That is, testing whether one feature's relative abundance slope for a metadatum is different from the median relative abundance slope for that metadatum is the same as testing whether that feature's absolute abundance slope is different from 0. This motivates the median comparison test implemented in MaAsLin 3.

Covariate slopes are unaffected by extraction efficiency

Now, let the extraction efficiency differ by feature, but assume each feature's extraction efficiency depends only on the feature, not on what else is in the sample. Let A_{ik} and Z_{ik} be the true absolute and log absolute abundances as above, and let E_i be the sampling efficiency of feature i with $S_i = \log_2(E_i)$. Now, let $T_k = \sum_i A_{ik}E_i$ and $D_k = \log_2(T_k)$, so $2^{D_k} = \sum_i 2^{Z_{ik}+S_i}$. Then, the relative abundances can be written as

$$P_{ik} = A_{ik}E_i/T_k \iff 2^{Y_{ik}} = 2^{Z_{ik}}2^{S_i}/2^{D_k} \iff Y_{ik} = Z_{ik}+S_i-D_k \iff Z_{ik} = Y_{ik}+D_k-S_i.$$

Let $\mathbf{S}_i$ be S_i repeated p times to match the dimensions of the feature-specific slopes. With an equivalent decomposition to before,

$$\hat{\boldsymbol{\beta}}_i = (\mathbf{X}^T\mathbf{X})^{-1}\mathbf{X}^T\mathbf{Z}_i = (\mathbf{X}^T\mathbf{X})^{-1}\mathbf{X}^T\mathbf{Y}_i + (\mathbf{X}^T\mathbf{X})^{-1}\mathbf{X}^T\mathbf{D} - (\mathbf{X}^T\mathbf{X})^{-1}\mathbf{X}^T\mathbf{S}_i = \hat{\boldsymbol{\beta}}_i^{\text{rel}} + \hat{\boldsymbol{\beta}}^{\text{tot}} - \hat{\boldsymbol{\beta}}_i^{\text{eff}}$$

where $\hat{\boldsymbol{\beta}}_i^{\text{eff}}$ is the regression of the $\log_2$ sampling efficiency on the covariates $\mathbf{X}$. However, since all the elements of $\mathbf{S}_i$ are equal, $\hat{\boldsymbol{\beta}}_i^{\text{eff}}$ will just be an intercept and then zeros for all the slopes corresponding to covariates (i.e., $\hat{\boldsymbol{\beta}}_i^{\text{eff}} = [\hat{\beta}_{i0}, 0, ..., 0]^T$). Thus, excluding the intercept, the same result as before holds: $\hat{\boldsymbol{\beta}}_i = \hat{\boldsymbol{\beta}}_i^{\text{rel}} + \hat{\boldsymbol{\beta}}^{\text{tot}}$. Therefore, the same results as above follow, justifying the median comparison technique and its equivalence to absolute testing when at least half the features are unassociated with a metadatum.

With sparsity

For simplicity, consider the equal extraction efficiency case again. Before, the coefficients could be decomposed as $\hat{\boldsymbol{\beta}}_i = \hat{\boldsymbol{\beta}}_i^{\text{rel}} + \hat{\boldsymbol{\beta}}^{\text{tot}}$ where $\hat{\boldsymbol{\beta}}^{\text{tot}}$ was the same for all features, but this was reliant on the data matrix $\mathbf{X}$ being the same for all features. When some features have zero abundances and only non-zero abundances are included in the regression, there will be different matrices $\mathbf{X}^{(i)}$ for each feature. Thus, the equation relating the slopes is $\hat{\boldsymbol{\beta}}_i = \hat{\boldsymbol{\beta}}_i^{\text{rel}} + \hat{\boldsymbol{\beta}}_i^{\text{tot}}$ with an index on the total abundance regression coefficient. Since $\hat{\boldsymbol{\beta}}_i^{\text{tot}}$ can now differ by feature, the ordering and spacing of the $\hat{\boldsymbol{\beta}}_i$ are not necessarily the same as of the $\hat{\boldsymbol{\beta}}_i^{\text{rel}}$. To proceed, note that the following quantities are being estimated with these regressions:

$$\boldsymbol{\beta}_i = E(\hat{\boldsymbol{\beta}}_i) = E((\mathbf{X}^T\mathbf{X})^{-1}\mathbf{X}^T\mathbf{Z}_i|\mathbf{X}, A_{ik} > 0\ \forall k)$$
$$\boldsymbol{\beta}_i^{\text{rel}} = E(\hat{\boldsymbol{\beta}}_i^{\text{rel}}) = E((\mathbf{X}^T\mathbf{X})^{-1}\mathbf{X}^T\mathbf{Y}_i|\mathbf{X}, A_{ik} > 0\ \forall k)$$
$$\boldsymbol{\beta}_i^{\text{tot}} = E(\hat{\boldsymbol{\beta}}_i^{\text{tot}}) = E((\mathbf{X}^T\mathbf{X})^{-1}\mathbf{X}^T\mathbf{D}|\mathbf{X}, A_{ik} > 0\ \forall k)$$

Since $\boldsymbol{\beta}_i = \boldsymbol{\beta}_i^{\text{rel}} + \boldsymbol{\beta}_i^{\text{tot}}$, we would retain the consistent ordering and spacing properties, at least in expectation, if

$$E((\mathbf{X}^T\mathbf{X})^{-1}\mathbf{X}^T\mathbf{D}|\mathbf{X}, A_{ik} > 0\ \forall k) = E((\mathbf{X}^T\mathbf{X})^{-1}\mathbf{X}^T\mathbf{D}|\mathbf{X}) \iff E(\mathbf{D}|\mathbf{X}, A_{ik} > 0\ \forall k) = E(\mathbf{D}|\mathbf{X})$$

for all i. That is, the results before would hold if (1) all features are always present (no sparsity) or (2) the expected total abundance is independent of whether any particular

bioRxiv preprint doi: https://doi.org/10.1101/2024.12.13.628459; this version posted December 14, 2024. The copyright holder for this preprint (which was not certified by peer review) is the author/funder, who has granted bioRxiv a license to display the preprint in perpetuity. It is made available under aCC-BY 4.0 International license.

feature is actually present (i.e., the conditional expectation of $\mathbf{D}$ is the same with or without the conditioning on $A_{ik} > 0 \; \forall k$). When the carrying capacity of a community is determined by the covariates and the community is at carrying capacity (e.g., in an adult gut), this is more likely to be true. However, during periods of colonization (e.g., in the infant gut), this is unlikely to hold.

Test implementation

The test statistic is $\frac{\hat{\beta}_{ij}^{\text{rel}} - \hat{M}_j^{\text{rel}}}{\sqrt{\text{Var}\left(\hat{\beta}_{ij}^{\text{rel}} - \hat{M}_j^{\text{rel}}\right)}}$. The variance can be decomposed: $\text{Var}\left(\hat{\beta}_{ij}^{\text{rel}} - \hat{M}_j^{\text{rel}}\right) = \text{Var}\left(\hat{\beta}_{ij}^{\text{rel}}\right) + \text{Var}\left(\hat{M}_j^{\text{rel}}\right) - 2\text{Cov}(\hat{\beta}_{ij}^{\text{rel}}, \hat{M}_j^{\text{rel}})$. First, $\text{Var}\left(\hat{\beta}_{ij}^{\text{rel}}\right)$ is already estimated in the model fitting. Second, $\text{Var}\left(\hat{M}_j^{\text{rel}}\right)$ is estimated as $\frac{1}{4n(f_{\hat{\beta}_{ij}}(0))^2} = \frac{2\pi \widehat{\text{Var}_i(\hat{\beta}_{ij})}}{4n}$ based on the asymptotic distribution of the median assuming the $\hat{\beta}_{ij}$ are i.i.d. from a normal distribution with mean 0.[91] While this will almost always be violated practically, the estimate of the $f_{\hat{\beta}_{ij}}(0)$ will typically be too small (the estimated density will be too dispersed), resulting in a conservative rather than anti-conservative test. Third, $\text{Cov}(\hat{\beta}_{ij}^{\text{rel}}, \hat{M}_j^{\text{rel}})$ is estimated by bootstrapping the $\hat{\beta}_{ij}^{\text{rel}}$ from their approximate normal distributions and computing $\hat{M}_j^{\text{rel}}$. The computed test statistic is then evaluated against the distribution of $\hat{\beta}_{ij}^{\text{rel}}$ to determine a p-value. While the distribution will not be exact, it will typically be close to a standard normal.

bioRxiv preprint doi: https://doi.org/10.1101/2024.12.13.628459; this version posted December 14, 2024. The copyright holder for this preprint (which was not certified by peer review) is the author/funder, who has granted bioRxiv a license to display the preprint in perpetuity. It is made available under aCC-BY 4.0 International license.

1230 # Supplementary Figures

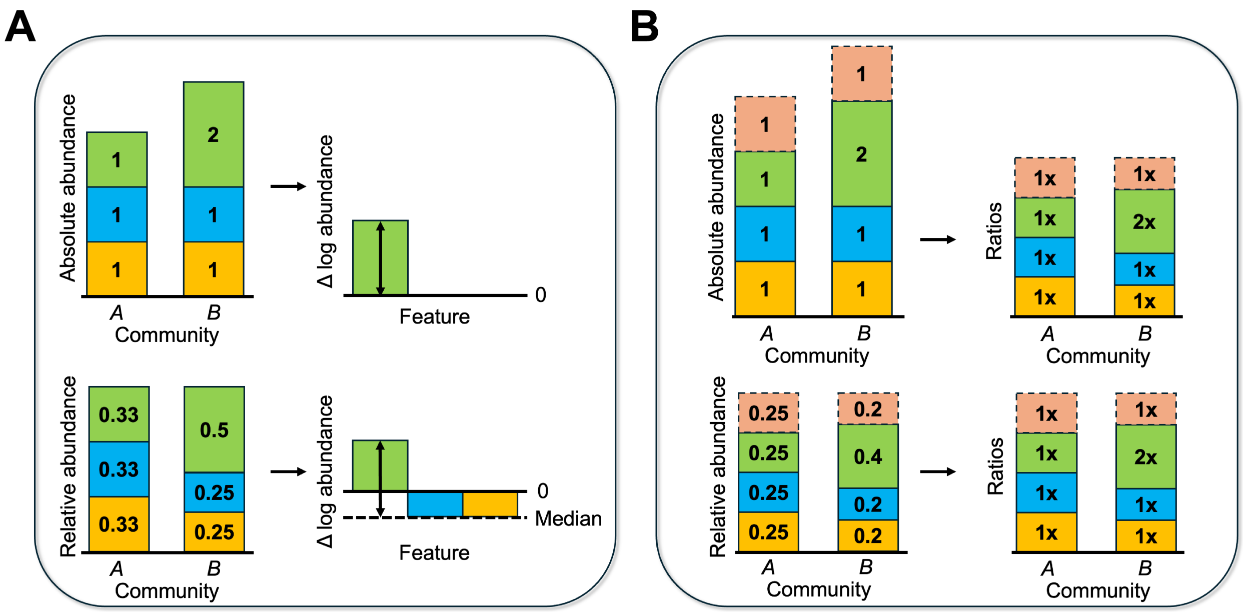

Supplementary Figure 1: MaAsLin 3 can account for compositionality with or without experimental protocol modifications. A. When only relative abundances are available, regressions are first fit on the relative abundance data, and the resulting coefficients are compared to the median of coefficients for each metadatum (median across the features). **B.** When spike-in abundances are known from the experimental protocol, the relative abundances are scaled to the spike-in to compute ratios. These ratios are then used in the regressions. Alternatively, the relative abundances can be scaled by a measure of total community abundance, producing estimated absolute abundances that are then used in the regressions.

bioRxiv preprint doi: https://doi.org/10.1101/2024.12.13.628459; this version posted December 14, 2024. The copyright holder for this preprint (which was not certified by peer review) is the author/funder, who has granted bioRxiv a license to display the preprint in perpetuity. It is made available under aCC-BY 4.0 International license.

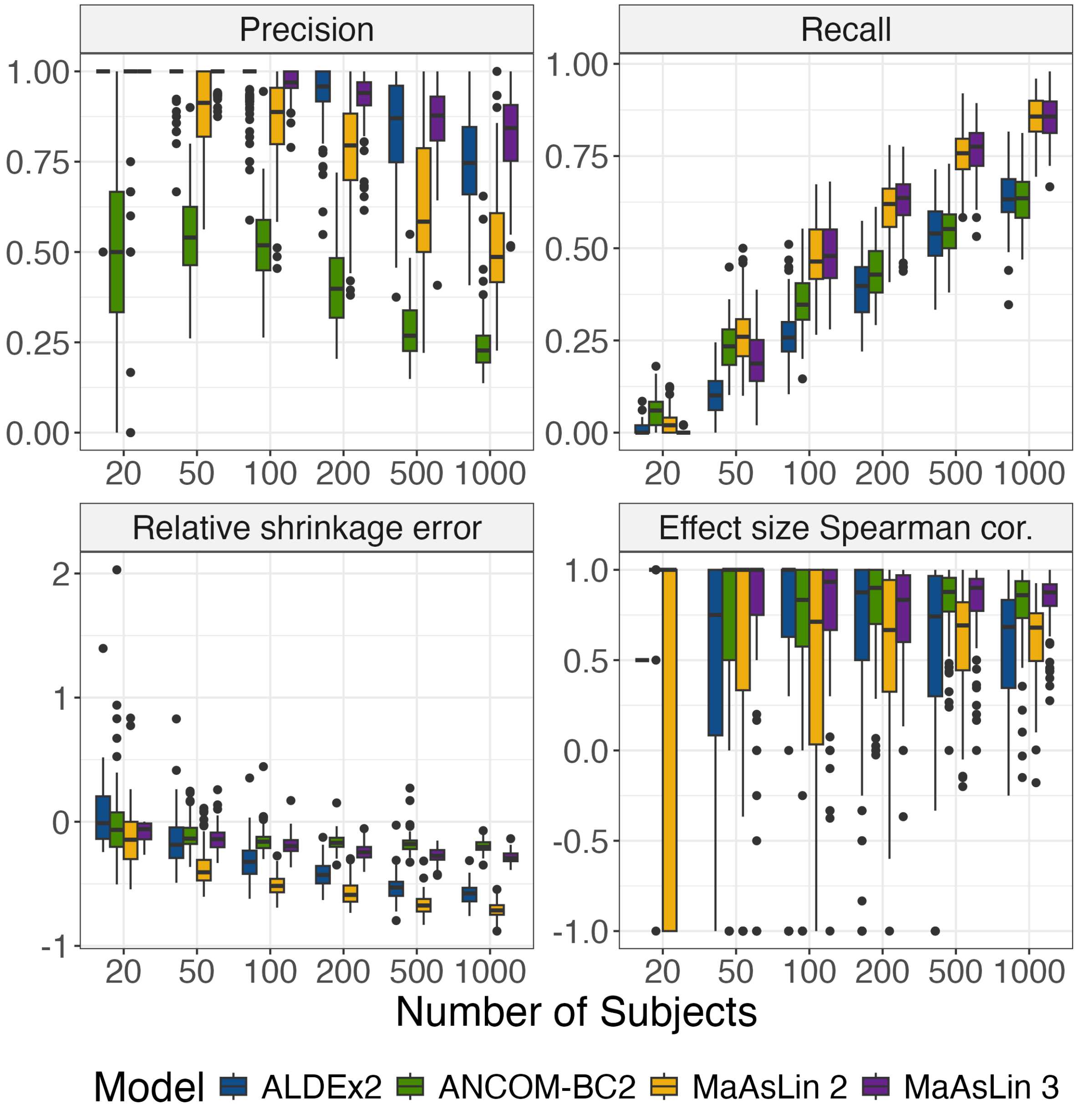

Supplementary Figure 2: MaAsLin 3 improves accuracy over other DA methods, particularly with high sample sizes. MaAsLin 3 and other common DA methods were run on the 100 synthetic log-normal datasets 2 from **Fig. 1C**. Each metric was calculated as before. 1 is optimal for all metrics except shrinkage, for which 0 is optimal. Each point represents a simulated dataset.

bioRxiv preprint doi: https://doi.org/10.1101/2024.12.13.628459; this version posted December 14, 2024. The copyright holder for this preprint (which was not certified by peer review) is the author/funder, who has granted bioRxiv a license to display the preprint in perpetuity. It is made available under aCC-BY 4.0 International license.

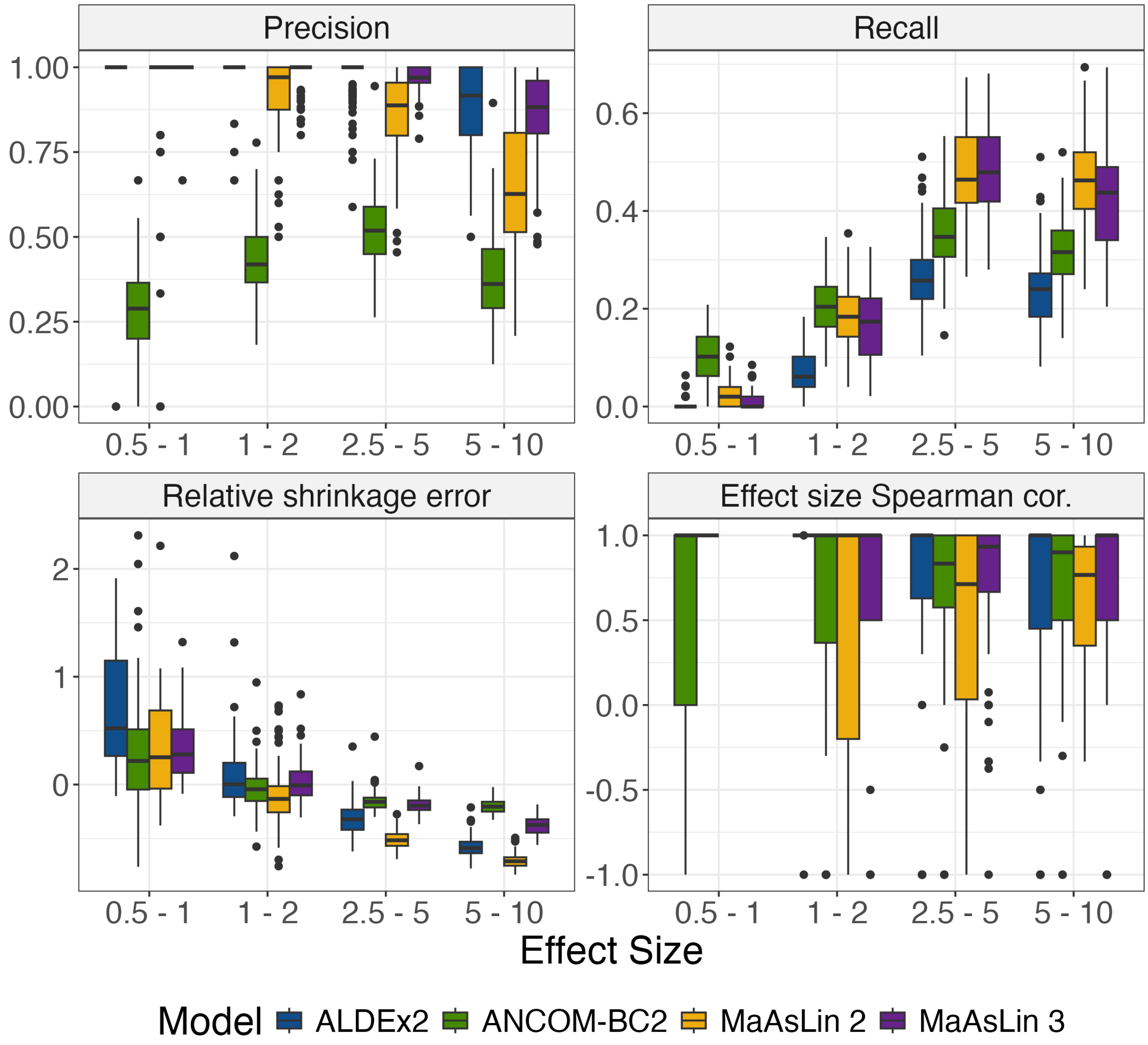

Supplementary Figure 3: MaAsLin 3 maintains high precision and accurate effect size estimation over a range of biologically relevant effect sizes. MaAsLin 3 and other common DA methods were run on 100 synthetic log-normal datasets from SparseDOSSA 2. The datasets were generated as in **Fig. 1C** but with 100 samples for all datasets and varying effect sizes. Each metric was calculated as before. 1 is optimal for all metrics except shrinkage, for which 0 is optimal. Each point represents a simulated dataset.

bioRxiv preprint doi: https://doi.org/10.1101/2024.12.13.628459; this version posted December 14, 2024. The copyright holder for this preprint (which was not certified by peer review) is the author/funder, who has granted bioRxiv a license to display the preprint in perpetuity. It is made available under aCC-BY 4.0 International license.

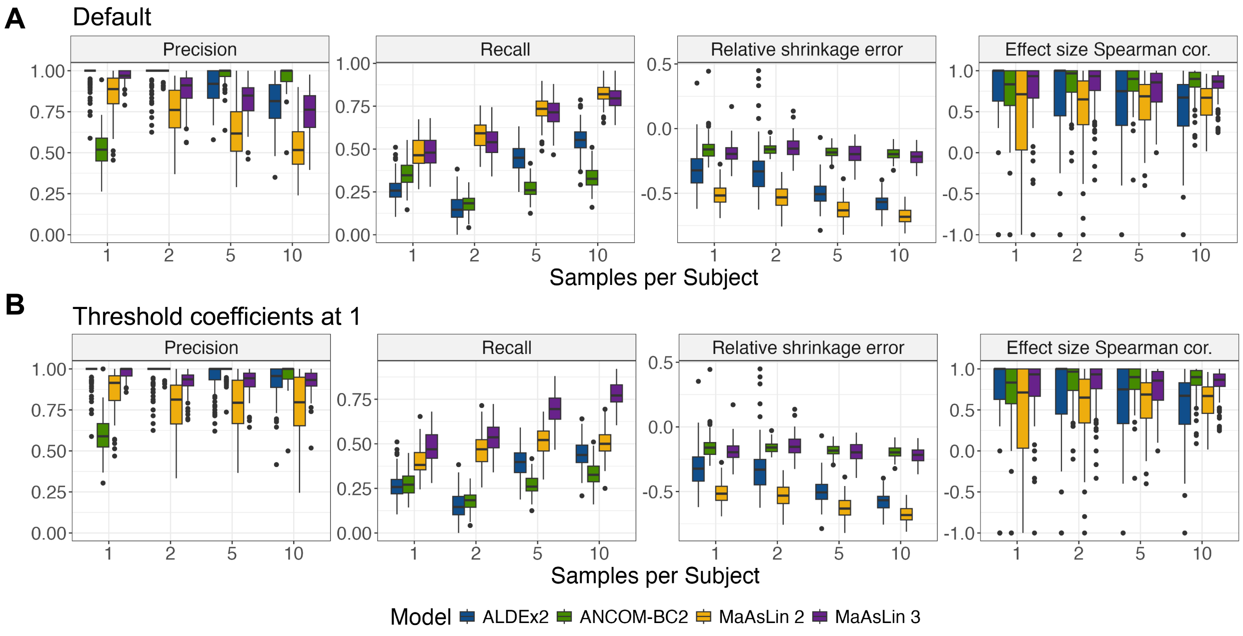

Supplementary Figure 4: MaAsLin models show high recall with repeated sampling at the cost of reduced precision, though precision can be substantially improved for MaAsLin 3 by thresholding fit coefficients. MaAsLin 3 and other common DA methods were run on 100 synthetic log-normal datasets from SparseDOSSA 2. Each dataset was generated as in **Fig. 1C** but with 100 subjects and varying numbers of samples per subject. For each feature, each subject was given a random intercept drawn from a normal distribution when generating the data. Each point represents a simulated dataset. The metrics were calculated as before on either all associations (**A**) or only associations with fit coefficients larger than 1 in absolute value (**B**). 1 is optimal for all metrics except shrinkage, for which 0 is optimal.

bioRxiv preprint doi: https://doi.org/10.1101/2024.12.13.628459; this version posted December 14, 2024. The copyright holder for this preprint (which was not certified by peer review) is the author/funder, who has granted bioRxiv a license to display the preprint in perpetuity. It is made available under aCC-BY 4.0 International license.

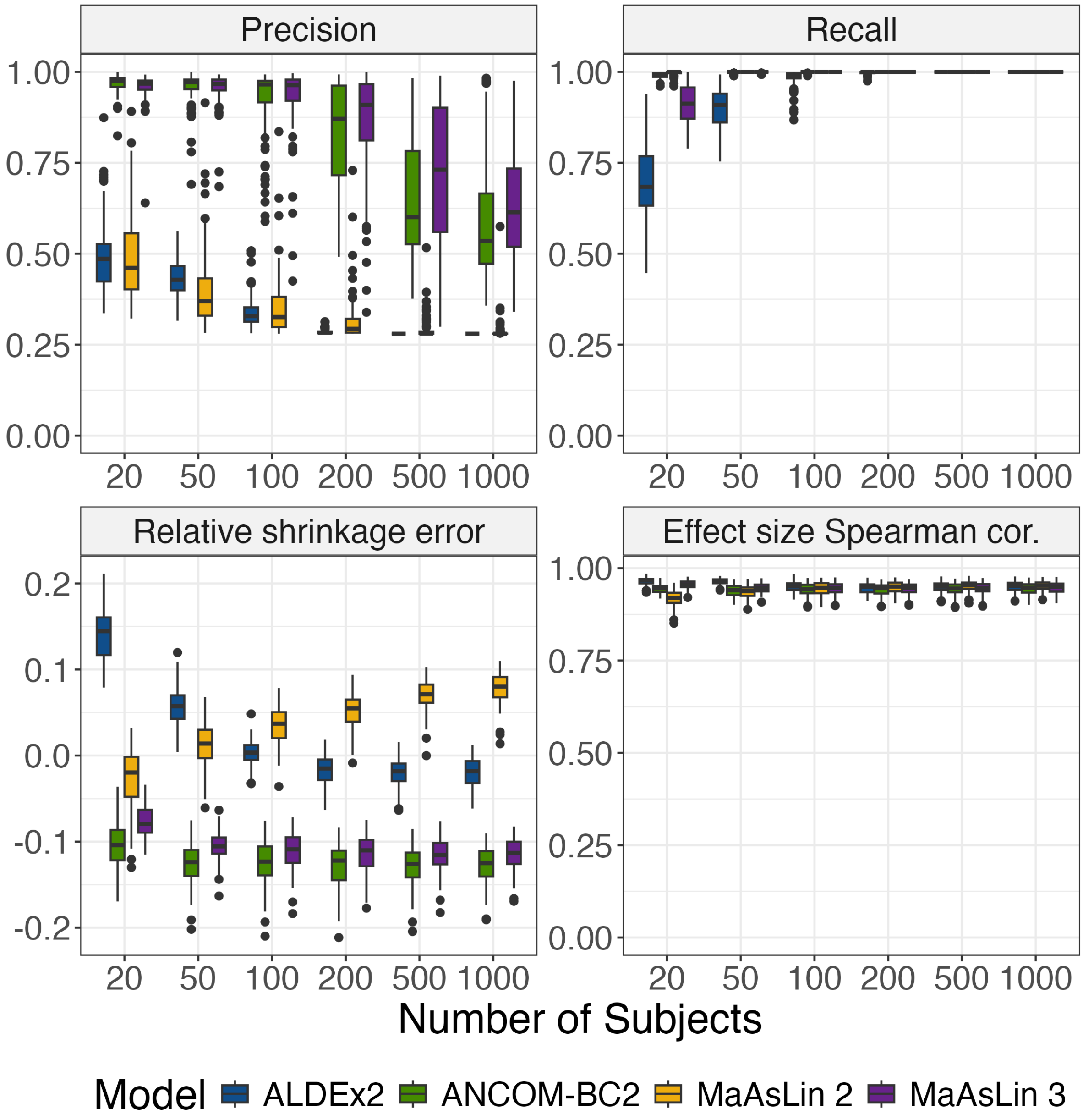

Supplementary Figure 5: MaAsLin 3 maintains or improves accuracy even when its modeling assumptions are violated. MaAsLin 3 and other common DA methods were run on 100 synthetic datasets generated with the 'soil' option of the ANCOM-BC evaluation. For these simulations, 1000 features and 2 groups were simulated with 10% of the feature-metadatum pairs having true associations with coefficients uniform from 2.5 to 5, half of which were positive and half of which were negative. Additionally, 20% of the features were set to have structural zeros in which all samples from one group lacked the feature. Highly skewed and unbalanced read depths (analogous to 16S read count) were drawn using the ANCOM-BC evaluation procedure with a mean depth of 50,000. Metrics were computed as before. 1 is optimal for all metrics except shrinkage, for which 0 is optimal. Each point represents a simulated dataset.

bioRxiv preprint doi: https://doi.org/10.1101/2024.12.13.628459; this version posted December 14, 2024. The copyright holder for this preprint (which was not certified by peer review) is the author/funder, who has granted bioRxiv a license to display the preprint in perpetuity. It is made available under aCC-BY 4.0 International license.

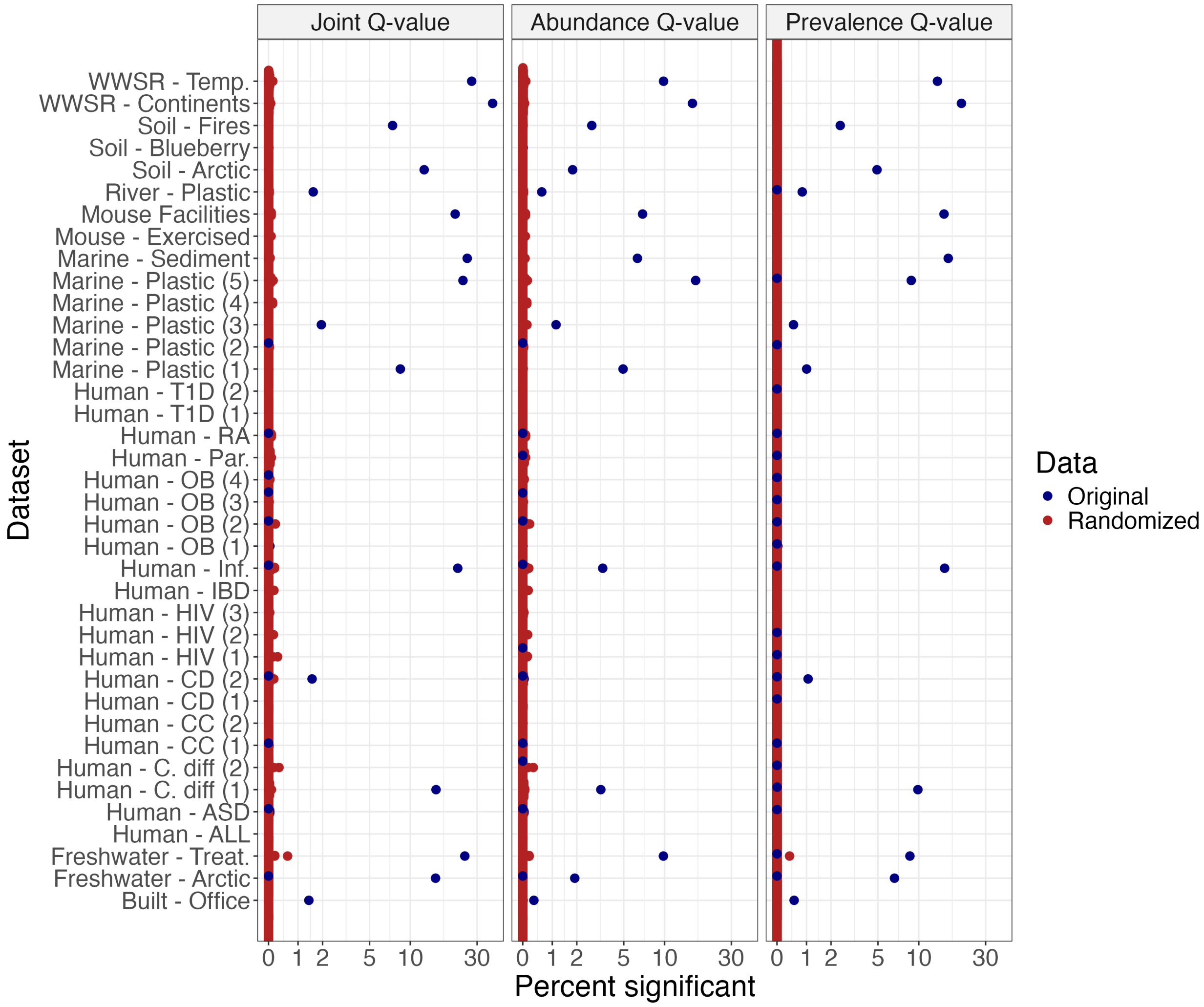

Supplementary Figure 6: A randomization test using real data shows that MaAsLin 3 almost never produces false positives when all associations are null. Using previous datasets,[1] 100 mock datasets were created for each real dataset by permuting the binary metadata labels. MaAsLin 3 was then run on the resulting randomized metadata and ASV tables, which should have no associations. For comparison, MaAsLin 3 was also run on the original datasets without randomization, which should have associations if they exist. The percent of significant feature-metadatum associations for both schemes is displayed. 0 is optimal for all randomized settings.

bioRxiv preprint doi: https://doi.org/10.1101/2024.12.13.628459; this version posted December 14, 2024. The copyright holder for this preprint (which was not certified by peer review) is the author/funder, who has granted bioRxiv a license to display the preprint in perpetuity. It is made available under aCC-BY 4.0 International license.

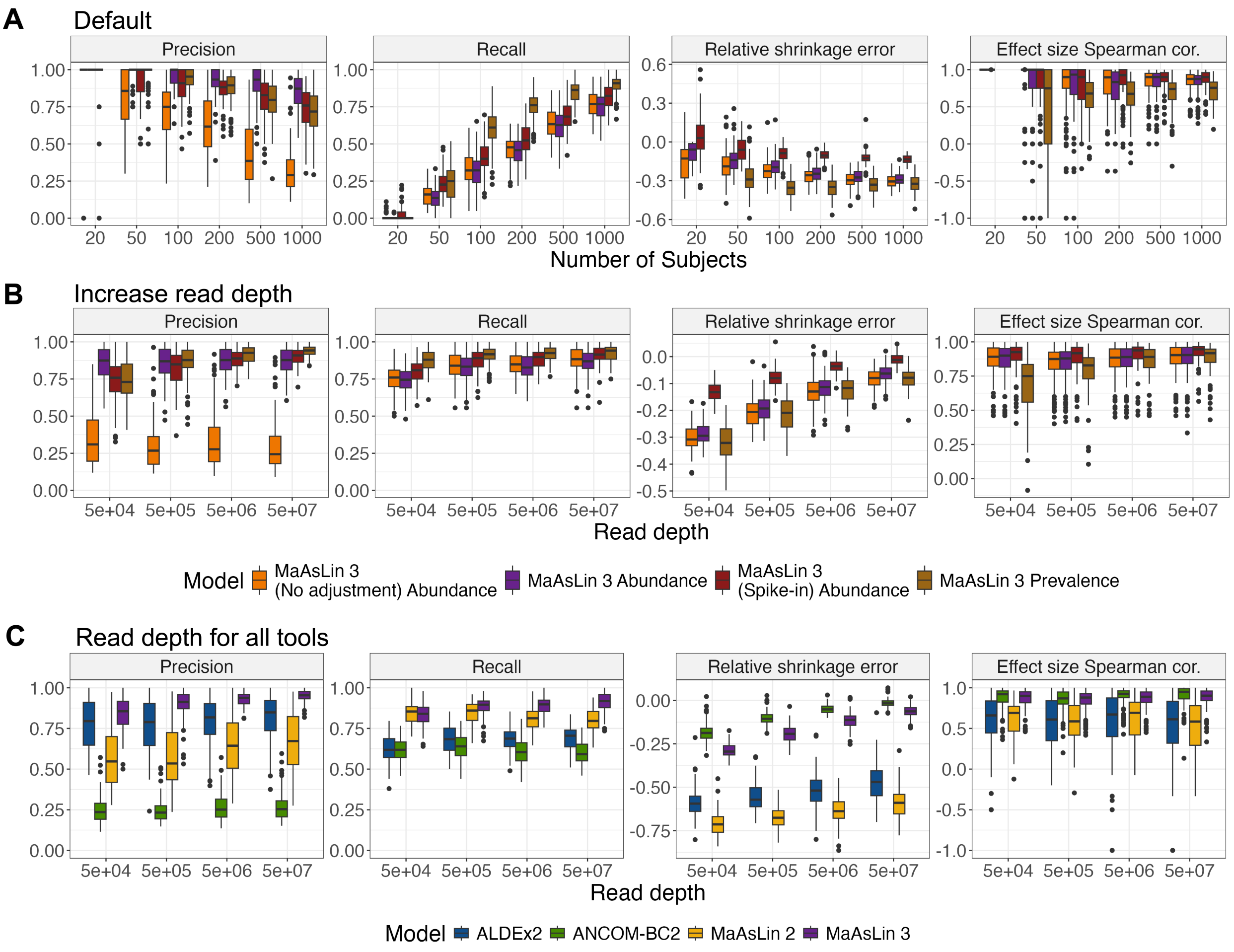

Supplemental Figure 7: Precision loss with high power can be mitigated by increasing read depth. A. Using 100 synthetic log-normal datasets from SparseDOSSA 2, MaAsLin 3 was run with no median adjustment for compositionality (abundance only), with the default adjustment (abundance only), with synthetic spike-in information (abundance only), and with the default prevalence setting. For **A**, the same datasets were used as for **Fig. 1C**. Significant associations (individual q-value less than 0.1) were only considered correct if they matched the true associations in the feature, metadatum, and type of association (prevalence/abundance). The relative shrinkage error and effect size correlation were computed as before. 1 is optimal for all metrics except shrinkage, for which 0 is optimal. Each point represents a simulated dataset. **B.** Datasets were generated as in A but holding the number of subjects fixed at 1000 and varying the mean read depth. **C.** The same metrics were evaluated for all methods on the datasets from B. In this evaluation, significant associations (q-value less than 0.1, joint q-value for MaAsLin 3) were considered correct if they matched the true associations in the feature and metadatum. A mismatch in association type—abundance versus prevalence—was allowed for all methods since no methods besides MaAsLin 3 report association type.

bioRxiv preprint doi: https://doi.org/10.1101/2024.12.13.628459; this version posted December 14, 2024. The copyright holder for this preprint (which was not certified by peer review) is the author/funder, who has granted bioRxiv a license to display the preprint in perpetuity. It is made available under aCC-BY 4.0 International license.

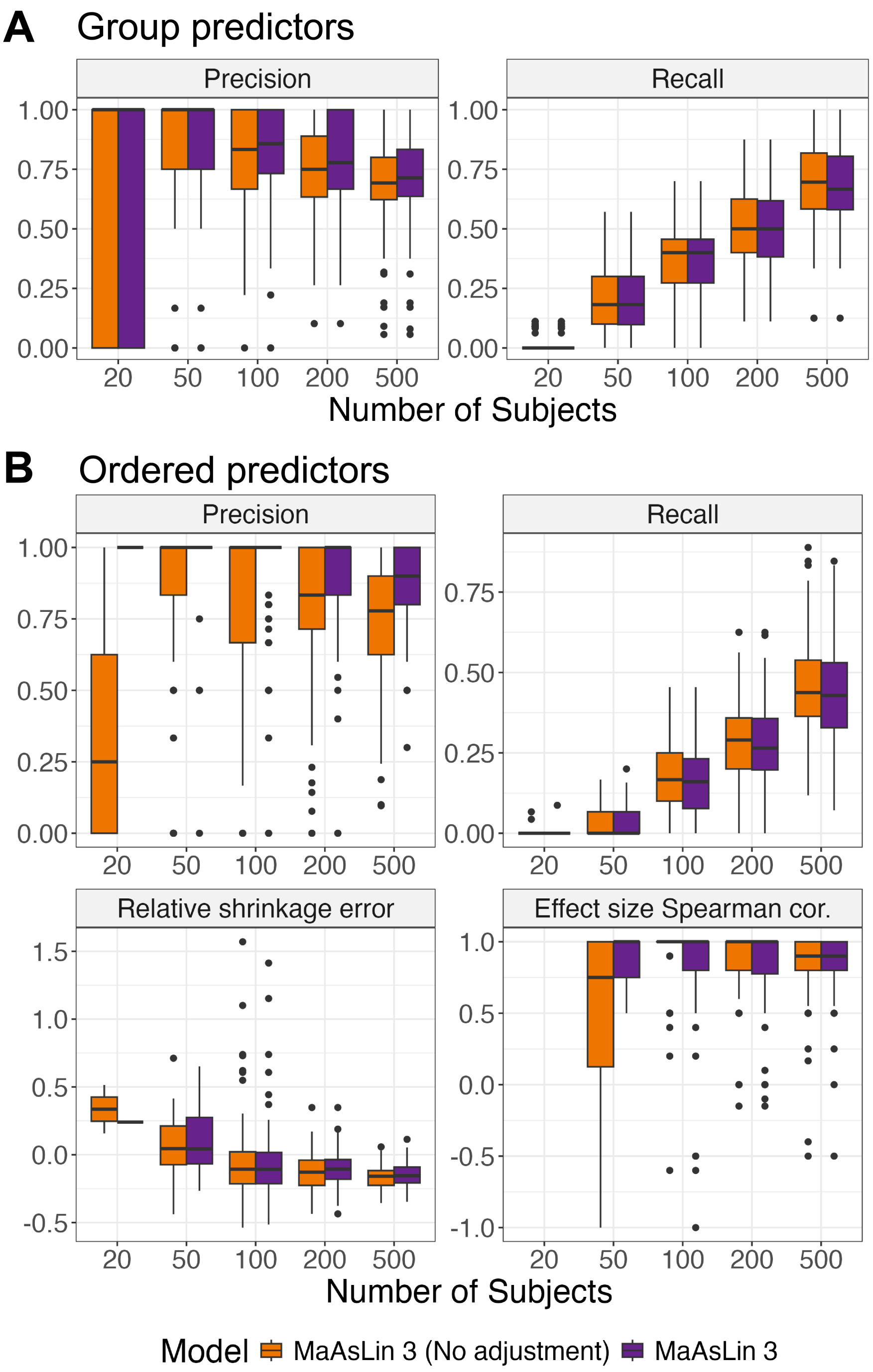

Supplementary Figure 8: MaAsLin 3 enables inference for group-wise differences and ordered predictors. MaAsLin 3 was run on 100 synthetic log-normal datasets from SparseDOSSA 2. For these simulations, 100 features and 2 metadata (one continuous or binary and one a categorical variable for group-wise or ordered predictors) were simulated with 10% of the feature-metadatum pairs having true associations. Coefficients for the continuous or binary variable were chosen from 2.5 to 5 uniformly. For the group-wise and ordered predictors, a value was chosen from 2.5 to 5 uniformly to represent the most extreme group's (level's) difference from baseline, and this value was subdivided according to a Dirichlet(1) distribution to obtain the coefficients for the other groups (levels). Half of the associations were positive; the rest were negative. Half were abundance associations; the rest were prevalence associations. The read depth (analogous to 16S read count) per sample was drawn from a log-normal distribution with a mean of 50,000. Significant associations (q-value less than 0.1, joint q-value for MaAsLin 3) were considered correct if they matched the true associations in the feature, metadatum, and type of association (prevalence/abundance). The metrics were calculated as before. 1 is optimal for all metrics except shrinkage, for which 0 is optimal. Each point represents a simulated dataset. Only the group predictor (**A**) or ordered predictor (**B**) variables were evaluated for accuracy. MaAsLin 3 was run with and without the median compositionality adjustment in both cases.

bioRxiv preprint doi: https://doi.org/10.1101/2024.12.13.628459; this version posted December 14, 2024. The copyright holder for this preprint (which was not certified by peer review) is the author/funder, who has granted bioRxiv a license to display the preprint in perpetuity. It is made available under aCC-BY 4.0 International license.

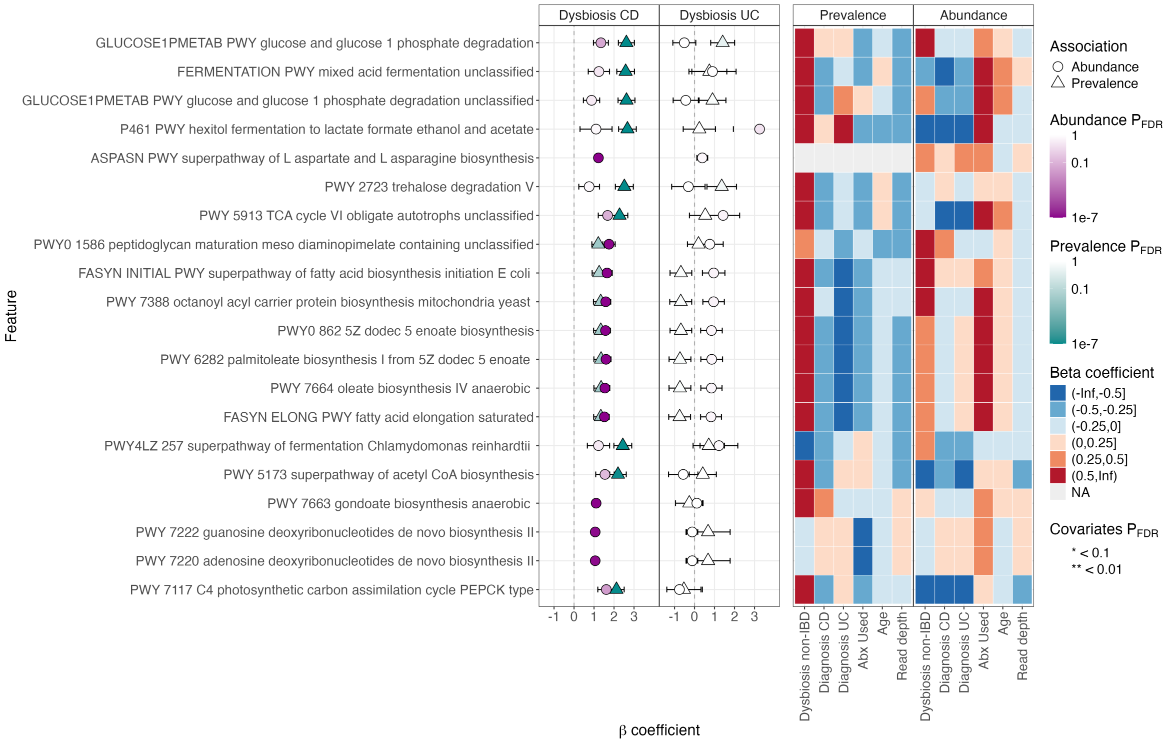

Supplementary Figure 9: MaAsLin 3 applied to the HMP2 metatranscriptomics data verifies and extends previous findings. The metatranscriptomics pathway abundances from the HMP2 cohort were regressed in MaAsLin 3 using a model that incorporated disease-stratified dysbiosis, disease diagnosis, antibiotic usage, age, read depth, a per-participant random intercept, and the pathway's metagenomic abundance as a feature-specific covariate.

bioRxiv preprint doi: https://doi.org/10.1101/2024.12.13.628459; this version posted December 14, 2024. The copyright holder for this preprint (which was not certified by peer review) is the author/funder, who has granted bioRxiv a license to display the preprint in perpetuity. It is made available under aCC-BY 4.0 International license.

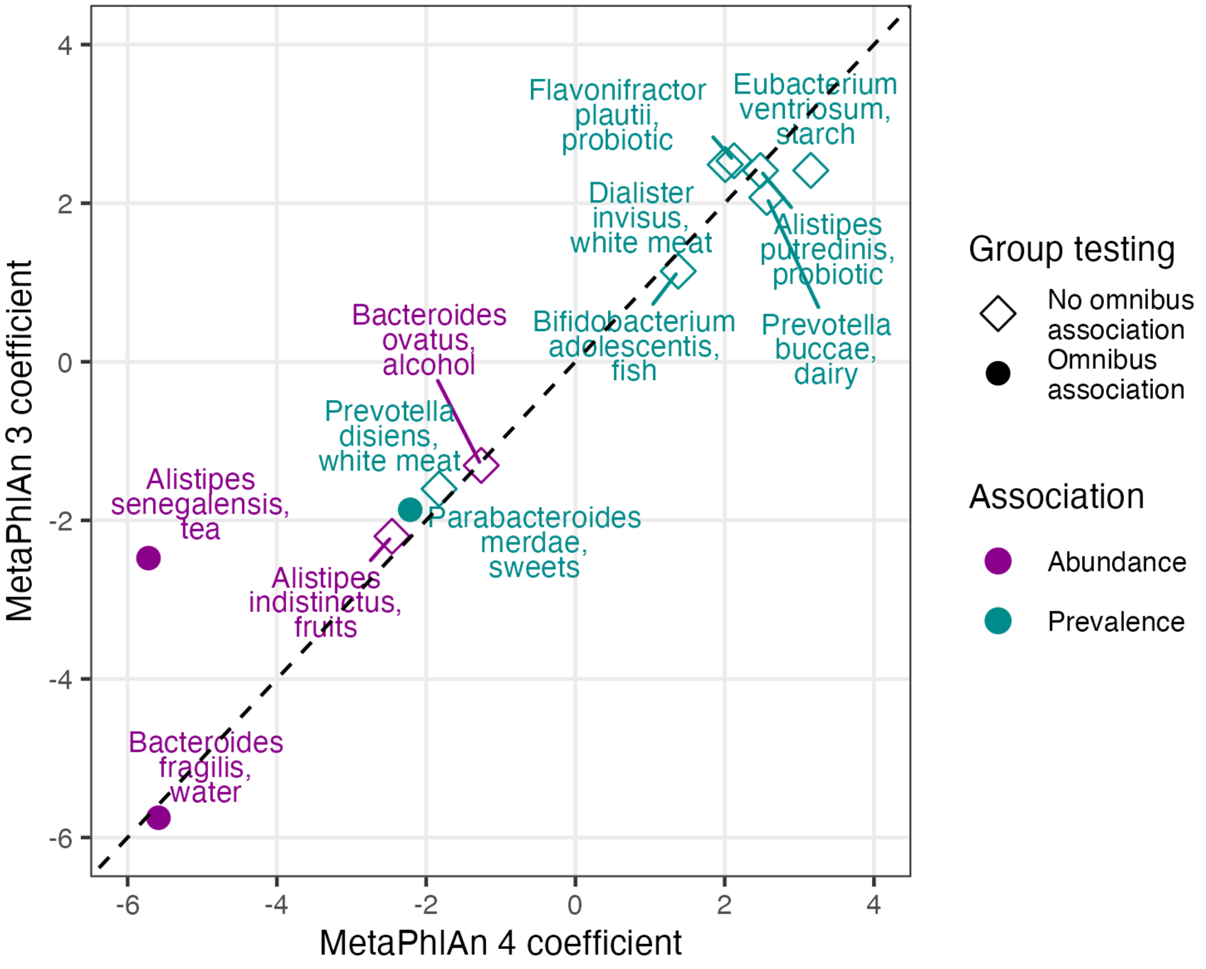

Supplementary Figure 10. Significant associations in both MetaPhlAn 3 and MetaPhlAn 4 profiles largely agree in fit coefficients. Using the subset of HMP2 participants with CD, abundances were regressed in MaAsLin 3 using a model that incorporated categorical dietary frequency information as a group or ordered predictor along with dysbiosis, antibiotic usage, age, read depth, and a per-participant random intercept. Associations identified as significant using both the MetaPhlAn 3 and MetaPhlAn 4 profiles are plotted.

bioRxiv preprint doi: https://doi.org/10.1101/2024.12.13.628459; this version posted December 14, 2024. The copyright holder for this preprint (which was not certified by peer review) is the author/funder, who has granted bioRxiv a license to display the preprint in perpetuity. It is made available under aCC-BY 4.0 International license.

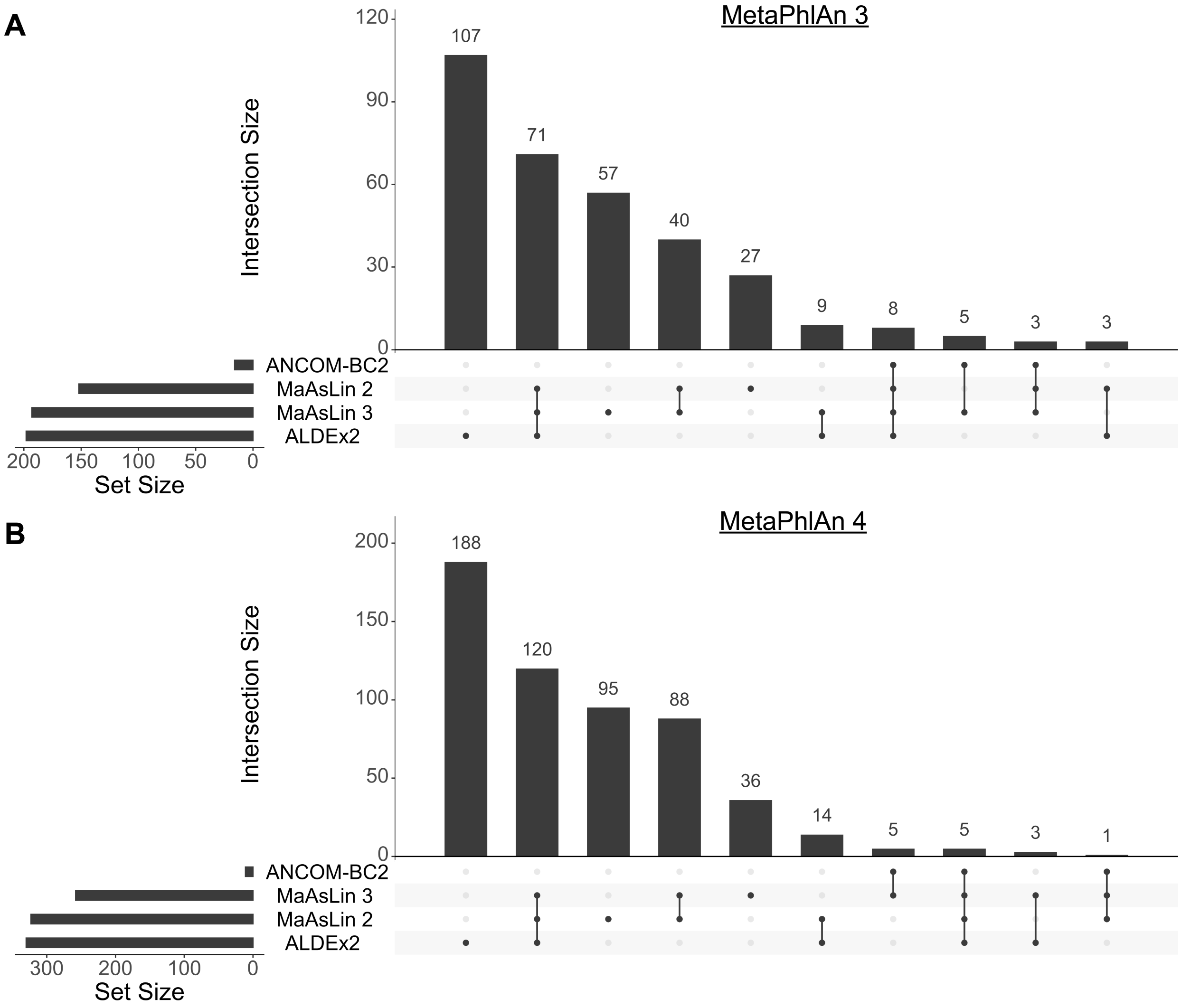

Supplementary Figure 11: Most IBD associations discovered by MaAsLin 3 overlapped with other methods. The species-level abundances from the HMP2 cohort profiled with MetaPhlAn 3 (**A**) or MetaPhlAn 4 (**B**) were regressed in each method using a model that incorporated disease-stratified dysbiosis, disease diagnosis, antibiotic usage, age, read depth, and a per-participant random intercept (or, for ALDEx2, a fixed intercept subsequently removed from analysis). Because of the possibility for false positives identified in the simulations, only significant (q-value ¡0.1) coefficients with absolute values greater than 1 were evaluated for their overlap.
